# Supplementary figures and images for: Candida albicans selection for human commensalism results in substantial within-host diversity without decreasing fitness for invasive disease
Source: PLoS Biol. 2023 May 19;21(5):e3001822. doi: 10.1371/journal.pbio.3001822 (PMC10234564; doi:10.1371/journal.pbio.3001822)

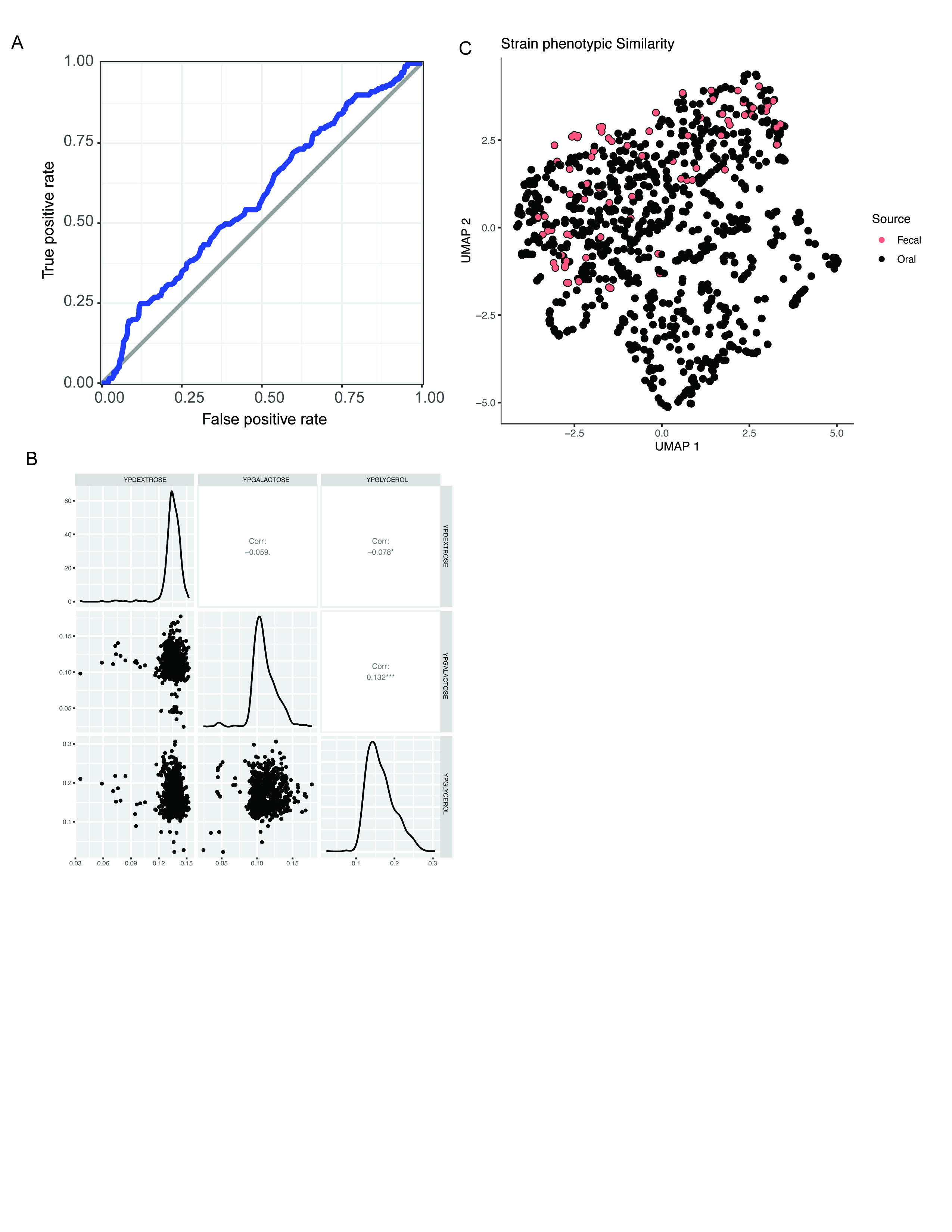

Supplement: S1 Fig — (A) Enrichment of fecal vs. oral samples by carrying capacity. (B) Correlation between growth rates in different carbon sources. (C) UMAP plot for strain phenotypic similarity. All growth conditions and invasion phenotypes were nonlinearly projected into 2D space as in Fig 1F, but this time colored by sample site. (TIF) [file pbio.3001822.s007.tif]

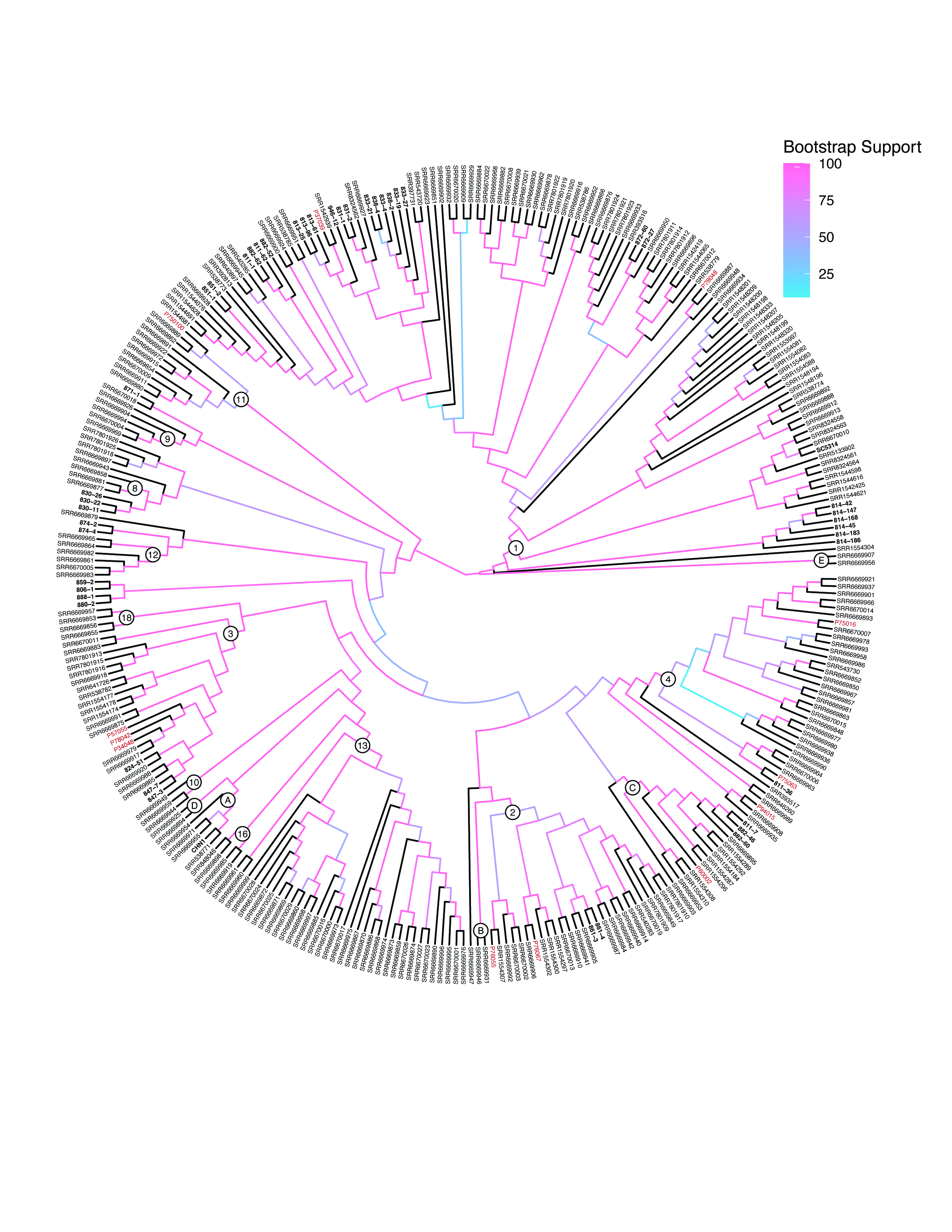

Supplement: S2 Fig — One hundred bootstrap replicates were generated by randomly sampling rows from the SNP table with replacement to yield a table with the same dimensions as the original SNP table. Distance matrices and neighboring-joining trees were generated for bootstrap replicates identically to the original SNP table. Bootstrap support values were computed based on the bootstrap replicate trees and annotated onto the original SNP tree with IQTree2. Bloodstream isolates used in Fig 4 are indicated with red text. (TIF) [file pbio.3001822.s008.tif]

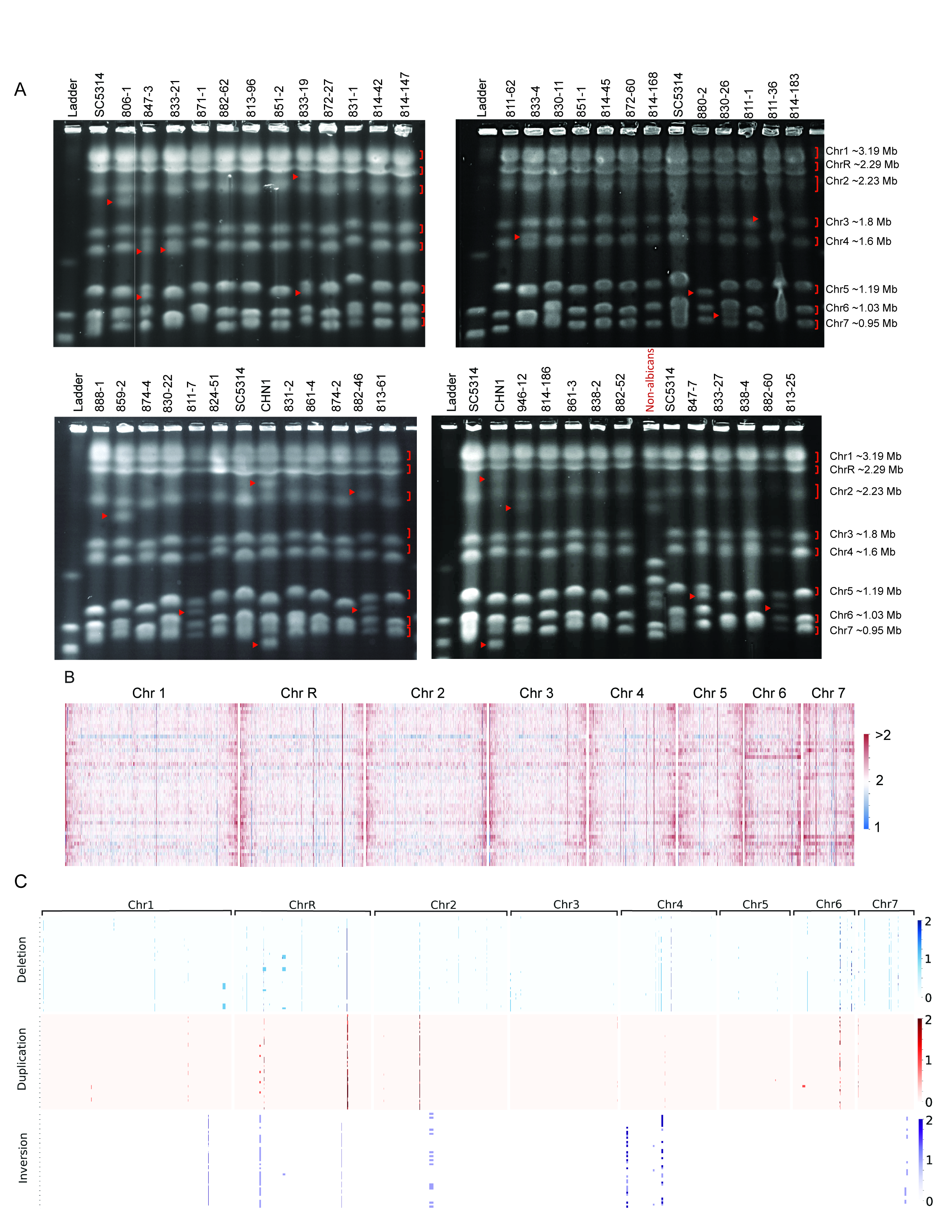

Supplement: S3 Fig — (A) CHEF karyotype gels were performed on the condensed set of C. albicans isolates and visualized using ethidium bromide staining. Red arrows indicate large chromosome banding patterns that differ from the SC5314 reference strain. Chromosome 5 showed especially extensive variability in size between isolates. (B) Heatmaps of read coverage across the condensed set of isolates for each chromosome. Each column represents a 500 bp bin of the reference genome and each row is an isolate from the condensed set. Values greater than 1 (red) suggest potential duplications while values less than 1 (blue) suggest potential deletions. Each row represents an isolate, with SC5314 represented by the top row. The rows are ordered, top to bottom, in the phylogenetic order used in Fig 3. (C) Heatmaps of Lumpy Structural variation calls for the condensed set of isolates. Each column represents a 500 bp bin of the reference genome and each row of the heat map for a given variant class is an isolate from the condensed set. The value for each bin indicates the number of SV calls overlapping the 500 bp window. (TIF) [file pbio.3001822.s009.tif]

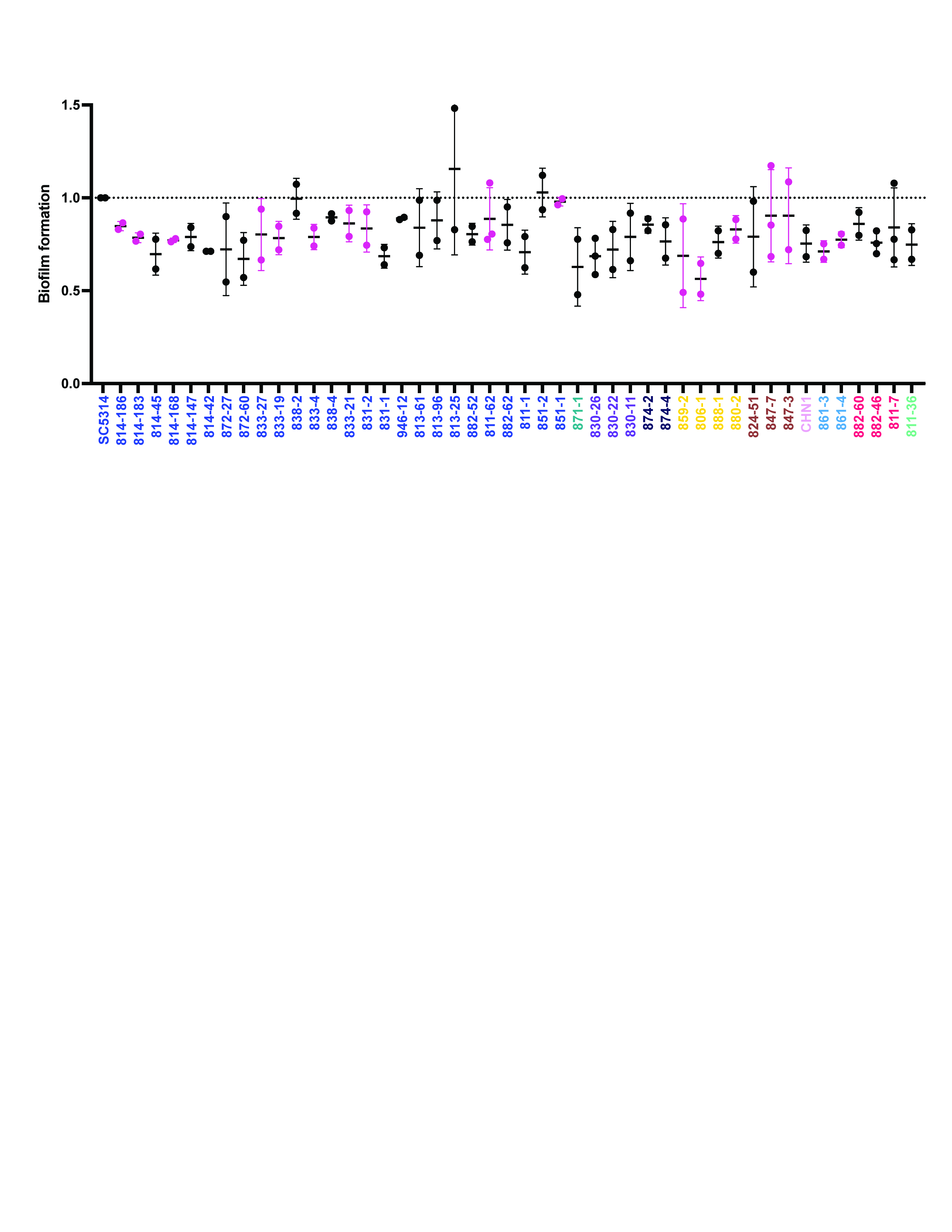

Supplement: S4 Fig — Each condensed set isolate was tested for its ability to form a biofilm on a plastic surface. Isolate labels were colored based on the nearest defined cluster from Fig 2 and ordered based on phylogeny. Black data points indicate an oral isolate and pink data points indicate a fecal isolate. Asterisks indicate P < 0.05 (*) compared with SC5314, one-way ANOVA compared with SC5314, with Dunnett’s post hoc test for multiple corrections. (TIF) [file pbio.3001822.s010.tif]

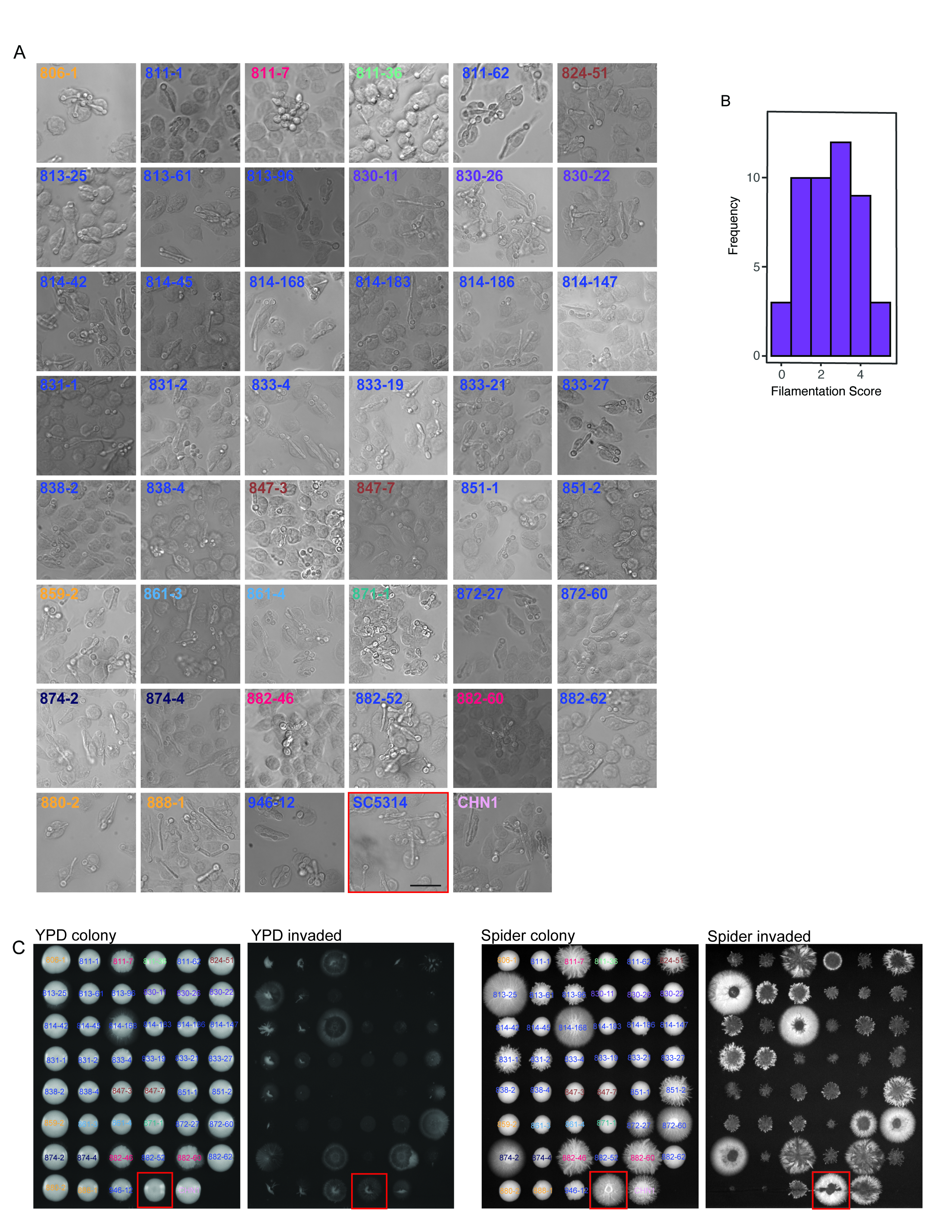

Supplement: S5 Fig — (A) Images of C. albicans condensed set isolates after growth in macrophages for 4 h. C. albicans were stained with calcofluor white. Images taken using DIC and DAPI channels at 20× magnification. Scale = 50 μM. (B) Histogram of the distribution of macrophage filamentation scores of the condensed set. (C) Colony morphology and agar invasion for C. albicans condensed set isolates on YPD agar at 30°C and Spider agar at 37°C after 5 days. (TIF) [file pbio.3001822.s011.tif]

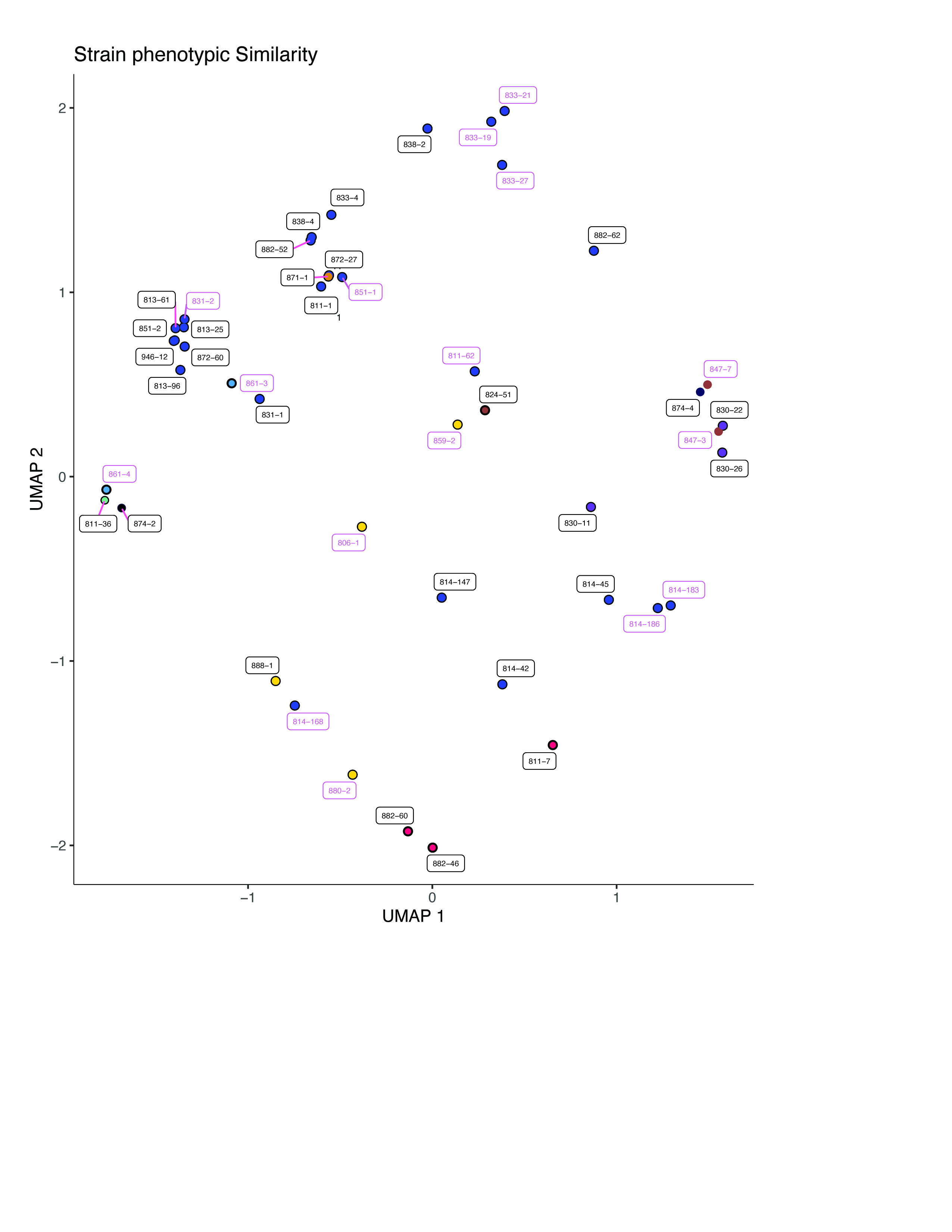

Supplement: S6 Fig — Nonlinear embedding dimensionality reduction was performed on the phenotypic data on the condensed set of isolates using UMAP. Clusters did not segregate by sample site, donor, or clade. Data used to generate the UMAP are included in S5 Table, raw data in S1 Information. Clade information is indicated by color. Pink or black outlines indicate fecal of oral isolate, respectively. (TIF) [file pbio.3001822.s012.tif]

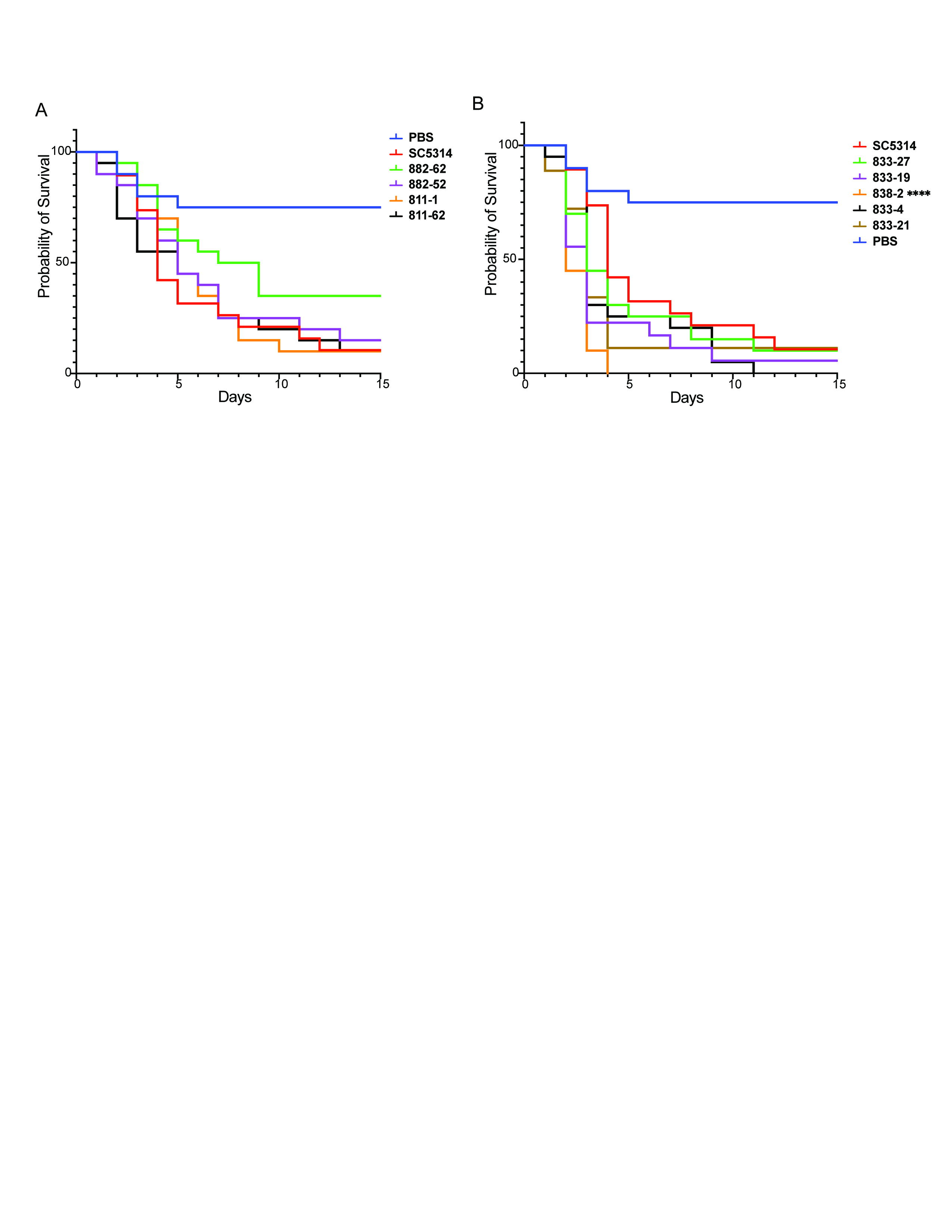

Supplement: S7 Fig — (A) Survival assays in G. mellonella, comparing the SC5314 reference to 4 isolates from donors 882 and 811. (B) Survival assays in G. mellonella, comparing the SC5314 reference to 5 isolates from donors 833 and 838. Each strain was standardized to 2 × 10^6^ cells/mL before inoculating 20 G. mellonella larvae per strain with 50 μL of prepared inoculum. Larvae were monitored daily for survival. Statistical differences were determined using a Mantel–Cox log-rank test. ** indicates P-value < 0.01, * indicates P-value < 0.05. (TIF) [file pbio.3001822.s013.tif]

Fig 3C raw blot

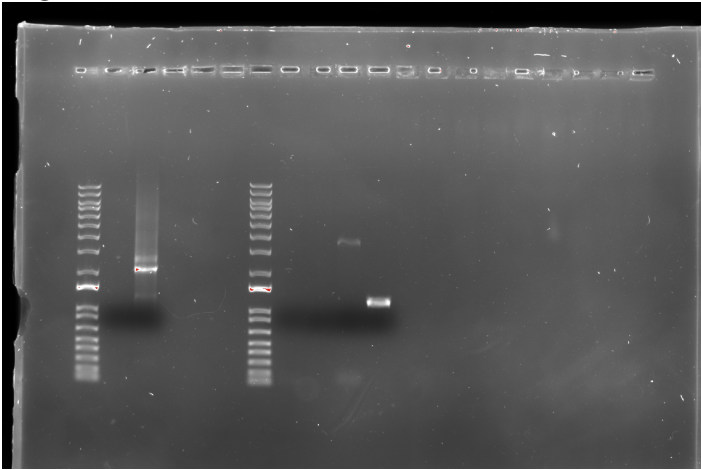

S3 Fig raw blots

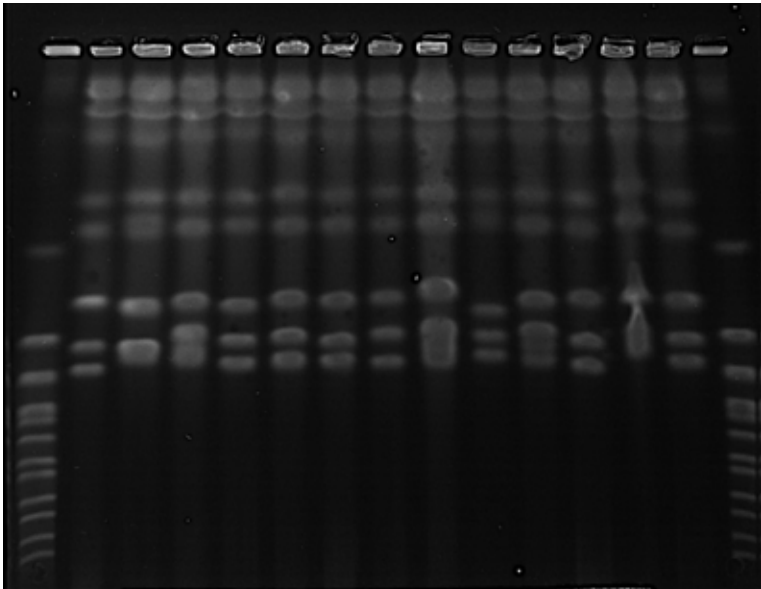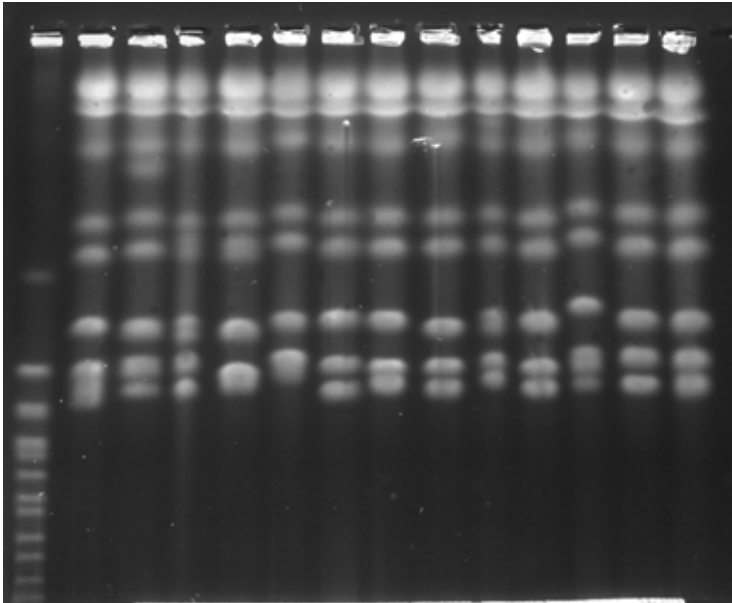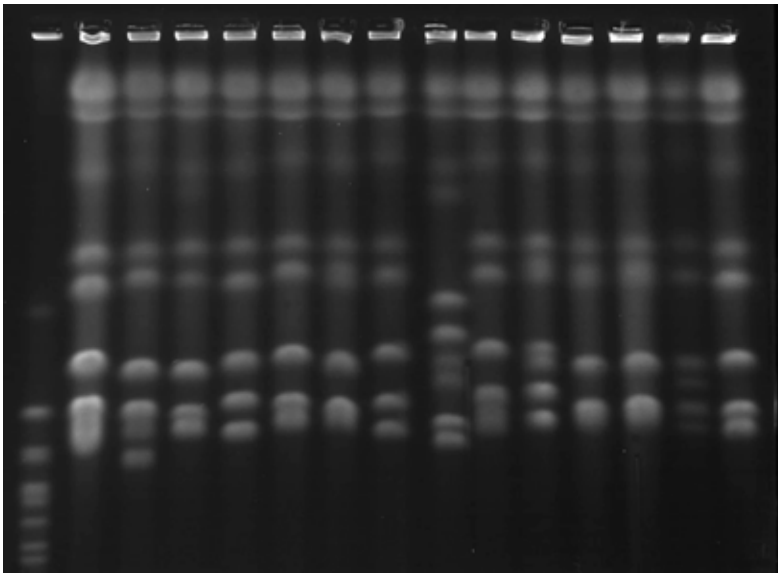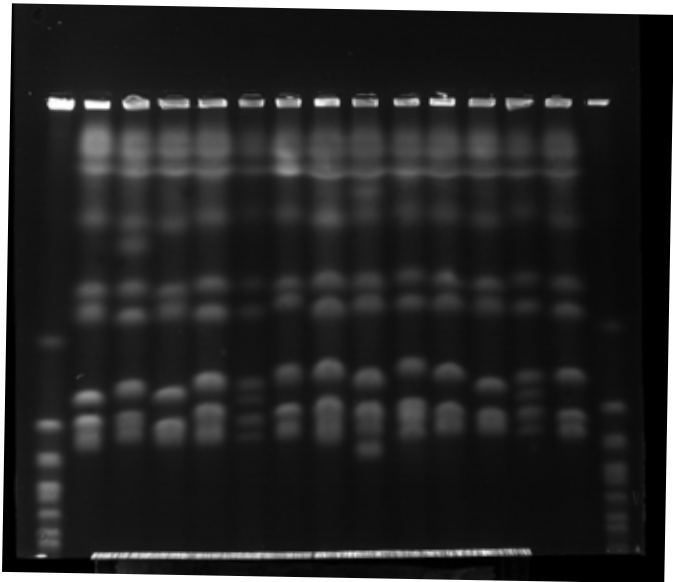

Supplement: S1 Raw Images — (PDF) [file pbio.3001822.s015.pdf]
